# *Smart Walk*: A Culturally Tailored Smartphone-Delivered Physical Activity Intervention for Cardiometabolic Risk Reduction among African American Women

**DOI:** 10.3390/ijerph20021000

**Published:** 2023-01-05

**Authors:** Rodney P. Joseph, Michael Todd, Barbara E. Ainsworth, Sonia Vega-López, Marc A. Adams, Kevin Hollingshead, Steven P. Hooker, Glenn A. Gaesser, Colleen Keller

**Affiliations:** 1 Center for Health Promotion and Disease Prevention, Edson College of Nursing and Health Innovation, Arizona State University, 500 N 3rd St., Phoenix, AZ 85004, USA; 2 College of Nursing and Health Innovation, Arizona State University, 500 N 3rd St., Phoenix, AZ 85004, USA; 3 School of Kinesiology, Shanghai University of Sport, Shanghai 200438, China; 4 College of Health Solutions, Arizona State University, Phoenix, AZ 85004, USA; 5 Southwest Interdisciplinary Research Center, Arizona State University, Phoenix, AZ 85004, USA; 6 College of Health and Human Services, San Diego State University, San Diego, CA 92182, USA

**Keywords:** exercise, physical activity, Black women, women’s health, health disparities, health equity, cardiovascular disease, type 2 diabetes, health promotion, mHealth

## Abstract

This article reports the results of *Smart Walk*: a randomized pilot trial of an 8-month culturally tailored, smartphone-delivered physical activity (PA) intervention for African American women with obesity. Sixty participants (age range = 24–49 years; BMI range = 30–58 kg/m^2^) were randomized to the *Smart Walk* intervention (n = 30) or a wellness comparison intervention (n = 30). Results supported the acceptability and feasibility of the intervention, as demonstrated by participant retention (85% at 4 months and 78% at 8 months), *Smart Walk* app use, and intervention satisfaction (i.e., 100% of PA participants completing the intervention [n = 24] reported they would recommend it to friend). *Smart Walk* participants also reported greater increases in moderate-to-vigorous PA (4-month between-arm difference in change [b] = 43.3 min/week; *p* = 0.018; Cohen’s *d* = 0.69; 8-month b = 56.6 min/week; *p* = 0.046; *d* = 0.63) and demonstrated clinically relevant, although not statistically significant (*p*-values > 0.05), baseline to 4 months improvements in cardiorespiratory fitness (b = 1.67 mL/kg/min; *d* = 0.40), systolic blood pressure (b = −3.33 mmHg; *d* = 0.22), diastolic blood pressure (b = −4.28 mmHg; *d* = 0.37), and pulse wave velocity (b = −0.46 m/s; *d* = 0.33). Eight-month cardiometabolic outcomes followed similar trends, but had high rates of missing data (45–53%) due to COVID-19 restrictions. Collectively, findings demonstrated favorable outcomes for acceptability and feasibility, while also highlighting key areas for refinement in future research.

## 1. Introduction

Regular aerobic physical activity (PA) improves numerous cardiometabolic risk factors, including high blood pressure [1,2,3], poor serum lipid profiles [4,5,6,7], insulin resistance [8,9], glucose intolerance [10,11], low cardiorespiratory fitness [12,13,14,15], and arterial stiffness [16,17,18]. PA has an inverse dose–response relationship with incident cardiovascular disease [19,20,21,22,23] and incident type 2 diabetes [22,24,25,26]. Likewise, regular aerobic PA promotes weight management and can assist with weight loss when combined with dietary interventions [23,27,28].

Despite the established cardiometabolic health benefits of aerobic PA, only 27% to 40% of African American (AA) women meet national PA guidelines [29] (i.e., 150 min/week of moderate-intensity PA, 75 min/week of vigorous PA, or an equivalent combination of durations and intensities [30]). Low PA levels among AA women contribute to their high burden of cardiometabolic diseases, as 57% of adult AA women are with obesity [31], 59% have cardiovascular disease [32], and 13% have been diagnosed with diabetes [33]). The prevalence of these conditions are higher than rates for White women (40%, 43%, and 8%, respectively), Hispanic women (44%, 43%, and 12%, respectively), and the average US population (42%, 48%, and 10%, respectively) [31,32,33]. Identifying effective interventions to increase PAamong AA women represents a public health strategy to improve cardiometabolic health disparities experienced by this population.

Culturally tailored PA interventions (i.e., interventions designed to recognize and address the unique sociocultural, norms, societal expectations, and behaviors of the intended population [34]) have consistently resulted in increased PA and improved health outcomes among AA women [35,36,37]. Such interventions represent a health equity approach to reducing PA-related health disparities among AA women and are a recommended strategy to reduce health disparities in the U.S. [38]. To date, most culturally tailored PA interventions for AA women have used in-person delivery strategies (e.g., small group or one-on-one counseling approaches) [35,36,37]. Few researchers have leveraged mobile or smartphone technology to deliver culturally tailored PA interventions to AA women despite their high level of smartphone ownership (i.e., 83% of AAs report owning a smartphone [39]) and the established effectiveness of mHealth PA interventions among non-AA populations (i.e., mHealth PA interventions, on average, increase PA by approximately 36 to 73 min/week [40,41,42]). A 2018 review [43] of electronic and mobile phone-delivered PA interventions among racial and ethnic minority women reported the paucity of research in this area by identifying only 3 studies (all pilots) testing mobile or smartphone interventions to promote PA among AA women. Two studies used text messaging [44,45] and one used both Facebook and text messaging [46]. Since this review, two additional studies have been published, one examining the efficacy of a smartphone app designed to promote light-intensity PA [47], and the other evaluating the feasibility of the smartphone-based PA intervention used for the current study, but in an older sample of AA women (aged 50–65) [48]. Findings of these studies have collectively indicated favorable outcomes for using mobile health (mHealth) technology to promote PA among AA women. However, limitations of prior mHealth PA interventions include infrequent use of appropriate control groups, limited assessment of PA-related health outcomes (including measures of cardiometabolic disease risk), and minimal use of cultural tailoring. Thus, highlighting the need for additional rigorously designed trials examining the utility of culturally tailored mHealth PA interventions among AA women. 

This paper reports the results of a randomized pilot trial evaluating *Smart Walk*, a culturally tailored, Social Cognitive Theory (SCT)-based smartphone-delivered intervention designed to increase PA and reduce cardiometabolic disease risk among insufficiently active AA women with obesity. The primary study aim was to examine the feasibility and acceptability of the intervention. Feasibility was assessed by examining recruitment and retention rates and the research team’s ability to deliver the intervention as intended. Acceptability was assessed by examining participant utilization of the smartphone app and self-reported intervention satisfaction. The secondary aim was to examine patterns of change in PA, cardiometabolic disease risk factors, and theory-based psychosocial constructs targeted by the intervention. We hypothesized that results would demonstrate favorably for acceptability and feasibility of the smartphone-delivered PA intervention and expected participants receiving the PA intervention to demonstrate greater improvements in PA and cardiometabolic disease risk factors as compared to the comparison group.

## 2. Materials and Methods

### 2.1. Study Design

An 8-month two-arm randomized pilot trial was conducted. Sixty African American women with obesity were assigned to receive the *Smart Walk* smartphone-delivered PA intervention or the *Smart Health* smartphone-delivered attention-matched wellness comparison condition. Months 1–4 consisted of the active intervention phase and months 5–8 included a no-contact follow-up period. The 4-month active intervention period was selected because it provides sufficient time for participants to increase their PA [49,50] and experience improvements in cardiometabolic risk factors [51,52]. The 4-month no-contact follow-up period was designed to evaluate the longer-term effects of the intervention after withdrawal of the active intervention. During the no-contact follow-up period, participants had access to their respective smartphone app; however, no new content was delivered. Data were collected at three time points: baseline, 4 months, and 8 months. Written informed consent was obtained from all study participants and study procedures were approved by the Institutional Review Board of Arizona State University (STUDY00006163). The study was also registered with clinicaltrials.gov (Identifier: NCT02823379). Readers are referred to a previous publication [53] for an in-depth description of the intervention’s study design, theoretical basis, and cultural tailoring. A brief description is included below.

### 2.2. Participants

Participants included 60 insufficiently active AA women with obesity aged 24–49 years. Specific inclusion criteria included: (a) self-identifying as an AA woman; (b) being between the ages 24 and 49 years; (c) having a BMI ≥ 30 kg·m^2^; (d) self-reporting 60 min/week or less of moderate-to-vigorous PA; and (e) having the ability to speak and read English. Exclusion criteria comprised: (a) participation in another PA, diet, or weight loss program at any time during the study; (b) contraindication to exercise based on the 2015 Physical Activity Readiness Questionnaire (PAR-Q+) [54], unless written physician consent for participation was provided by the participant’s physician; (c) pregnant at the time of screening or planned to become pregnant in the next 8 months; or (d) planned relocation outside of the Phoenix area within 12 months. 

### 2.3. Description of the Smart Walk PA Intervention

*Smart Walk* is a culturally tailored PA promotion intervention delivered through the *Smart Walk* smartphone app and text messages. The *Smart Walk* app included four key features: personal profile pages, culturally tailored video and text-based PA promotion modules, online discussion board forums, and a PA self-monitoring feature that integrated with Fitbit activity monitors. The behavioral goal was for participants to achieve 150 min per week of moderate-intensity aerobic PA [30]. Social Cognitive Theory (SCT) [55] served as the theoretical basis for the intervention, with constructs of behavioral capability, social support, self-efficacy, outcome expectations, and self-regulation underpinning study activities (see Table 2). Brief descriptions of each intervention component are below. See Joseph et al. [53] for screenshots of the *Smart Walk* app and a more detailed description of each intervention component.

Personal profile pages. This feature allowed participants to share brief biographical information (e.g., name, age, neighborhood) and a profile picture with other participants. 

Multi-media video and text-based PA promotion modules. These modules served as the primary method for delivering the intervention’s educational and behavioral content. Brief (3–7 min) video modules, each accompanied by print and image-based PA promotion materials, were narrated by the study spokesperson describing the week’s PA promotion topic (see Table 1). During months 1–3, new PA promotion module was delivered each week. In month 4, new modules focusing on post-intervention PA maintenance were delivered every two weeks. 

Discussion forums. Each PA promotion module was accompanied by a discussion board forum. These forums allowed participants to discuss module content, share PA-related personal experiences or thoughts, and provide/receive social support. Separate general “Community Board” and “Meet-up” forums allowed participants to discuss topics not related to module content and to arrange group exercise activities. 

**Table 2:** Social Cognitive Theory (SCT) constructs addressed by the *Smart Walk* intervention.

SCT Construct	Concepts Leveraged by the Intervention	Description of How the Intervention Targeted Each Theoretical Construct
**Behavioral Capability**—PA-related knowledge and skill	Knowledge Skill	Multi-media modules included content on: ○ Description of- and difference between PA and exercise ○ Differences between aerobic and. Muscle-strengthening PA ○ National PA guidelines ○ Types of PA that can be performed to achieve PA guidelines ○ How to gauge PA intensity.
**Social Support**—Extent to which family and friends support, encourage/discourage, and/or influence PA engagement.	Emotional Support (i.e., empathy, trust and caring) Appraisal Support (i.e., information for self-evaluation) Informational Support (i.e., advice/suggestions)	Multi-media modules and text messages: ○ Provided encouragement and empowerment for PA (i.e., emotional support) ○ Emphasized PA as a self-care (i.e., appraisal support) ○ Encouraged participants to reflect on the reasons they should be active (i.e., appraisal support) ○ Included strategies and tips to facilitate social support for PA from their family/friends (i.e., informational support) Discussion board prompts facilitated dialogue for participants to provide/receive emotional support for PA
**Self-efficacy**—Confidence in one’s ability to overcome barriers and be physically active	Mastery Experience (i.e., previous experiences with a behavior) Social Modeling (i.e., observing others perform a behavior) Verbal Persuasion (i.e., verbal encouragement) Emotional Arousal (i.e., improving emotional states by reducing stress/anxiety and encouraging of positive emotions)	Participants self-monitored their PA using the Fitbit and the activity tracking feature (i.e., mastery experiences) Multi-media modules: ○ Depicted AA women engaging in PA (social modelling) ○ Included PA testimonials from AA women (i.e., social modelling) ○ Reinforced the notion that PA does not have to be structured or difficult (emotional arousal) ○ Provided tips on how walking can be easily integrated into daily activities (i.e., emotional arousal) Multi-media modules and text messages included words of encouragement and empowerment (i.e., verbal persuasion) Discussion boards provided a forum for participants encourage each other to be physically active (i.e., verbal persuasion)
**Outcome Expectations**—Anticipated outcomes associated with PA engagement	Beliefs related to the likelihood, value, and consequences of a behavior	Weekly video and text modules illustrated the health and social benefits associated with regular PA, including: ○ Reduced risk for heart disease and type 2 diabetes ○ Weight management ○ Increased energy for daily activities ○ Improved quality and years of life.
**Self-regulation**—Management of intrapersonal and interpersonal processes to achieve PA goals	Self-monitoring Goal Setting Self-reward	Use of the Fitbit activity monitor and self-monitoring feature on the app to track PA Intervention materials encouraged creation of short and long-term PA goals and developing self-rewards for achieving PA goals.

PA Self-monitoring feature. To allow for PA self-monitoring, participants were given a Fitbit Inspire HR (Fitbit Inc., San Francisco, CA) activity monitor to wear during the study. Data collected by the Fitbit integrated with the *Smart Walk* application to allow participants to track their MVPA. A walking cadence of 100 steps/minute was used to define MVPA by the app [56,57]. 

PA promotion text messages. Participants were sent 3 PA promotion text messages each week during the active 4-month intervention period. Text messages included inspirational quotes, reminders, and tips for how to increase daily PA. 

### 2.4. Cultural Tailoring of the Intervention

Cultural tailoring was achieved through a comprehensives synthesis of the literature [58,59] and focus group research with local AA women [60,61,62]. Using Resnicow’s cultural tailoring framework, the intervention was tailored at surface and deep structure levels. Surface level cultural tailoring (i.e., packaging a health promotion to coincide with the intended population’s observable social and behavioral characteristics) [34], included use of statistics to illustrate PA and cardiometabolic disease health disparities among AA women, pictures of AA women engaging in PA throughout PA promotion materials, and having a member of the local AA community serve as the study spokesperson and deliver the intervention content via weekly video modules. Deep structure cultural tailoring (i.e., recognition of a group’s sociocultural norms, values, and behaviors) focused on key concepts collectivism/ethic of care, physical appearance norms (hair care/body shape concerns), and racial pride/role modeling (Table 3). 

### 2.5. Description of the Smart Health Attention-Matched Wellness Comparison Intervention

Women assigned to the smartphone-delivered attention-matched wellness comparison condition received a surface-level culturally tailored health promotion intervention, entitled *Smart Health*, focusing on health topics not related to PA and cardiometabolic disease risk (i.e., cancer prevention and screenings, skincare, and how to communicate with your healthcare provider; Table 1).This intervention was delivered using the same smartphone application platform as the PA intervention, with delivery following a similar timeline. The only difference between study groups was that the *Smart Health* app did not receive a PA tracking tool (see Table 4). 

### 2.6. Measures

#### 2.6.1. Demographic Characteristics

Demographic characteristics (i.e., age, income, relationship status) were collected using a survey developed for the current study. 

#### 2.6.2. Feasibility and Acceptability

Feasibility was assessed by examining participant recruitment and retention rates and the ability of the research team to deliver the intervention as intended. Acceptability was assessed by smartphone application use, Fitbit wear, and self-reported treatment acceptance using a post-intervention participant satisfaction survey. Specific details of these outcomes are below

Recruitment. Assessed by the duration of time (in months) to recruit our a priori sample of 60 participants.

Retention. Calculated as the proportion of enrolled participants who participated in 4- and 8-month study follow-up assessments. 

Objectively-measured App Use, Fitbit Wear, and Text Message Delivery.*Smart Walk* app use was assessed using built-in analytic tracking software. This software recorded when a participant loaded a PA module on their phone and when they posted in a discussion forum. Fitbit wear was assessed by the number of days participants wore the Fitbit. Following Hartman et al. [63], for a day to count as a valid wear day, a participants had to accumulate at least 1 min of light or moderate-intensity PA (as classified via Fitbit’s tracking algorithm) on a given day. Because participants were instructed to wear the Fitbit to track their activity, instead of wearing the device all day, achieving 1 min of activity indicated that the participant wore the device for part of the day. Twilio cloud-based communication platform was used to deliver study text messages. This platform recorded the time and date a text message was delivered to each participant. An error note was provided to the research staff if a text message was not delivered. 

Treatment Acceptance. Assessed at 4 months using a 37-item survey adapted from previous research [46,64]. This survey comprised multiple choice and open-ended questions about overall perceptions of the intervention, as well as thoughts on the specific intervention features (modules, activity tracker, discussion boards). 

#### 2.6.3. Physical Activity 

Self-reported PA and Energy Expenditure. Self-reported MVPA (min/week) was assessed by the Exercise Vital Sign Questionnaire [65], which measures frequency (days/week) and duration (min/day) of MVPA performed during the past week. MVPA min/week are calculated by multiplying frequency by duration. Weekly energy expenditure was measured by the 16-item REGICOR Short Physical Activity Questionnaire [66], a reliable and valid instrument that provides estimates of leisure-time energy expenditure, and minutes/week of sedentary time and time spent in light-, moderate-, and vigorous-intensity PA.

Accelerometer-measured PA. ActiGraph GT9X Link activity monitors were used to obtain an objective assessment of MVPA. Participants were instructed to wear the GT9X Link on their non-dominant wrist 24 h/day for 7 consecutive days. A valid assessment was defined as a participant wearing the device a minimum of 10 h/day on at least 4 days. Accelerometer data were collected in raw format at 30 Hz and downloaded to a computer (using a 60-s epoch length) for wear time verification using the Choi et al. [67] algorithm. After wear time was validated, raw accelerometer files were processed using the GGIR package [68] in R [69]. Minutes/day of MVPA performed in bouts of 1- and 10 min were estimated using algorithms developed by Hildebrand et al. (i.e., an acceleration threshold of 100 mg for moderate-intensity PA and a threshold of 428.8 mg for vigorous-intensity PA) [70].

#### 2.6.4. Cardiometabolic Risk Markers

Anthropometrics. Weight and height were measured in a private room with participants wearing light clothing and no shoes. Body weight was measured to the nearest 0.1 kg using a digital scale. Height was measured to the nearest 0.1 cm using a stadiometer. BMI was calculated as weight in kilograms divided by height in meters squared.

Cardiorespiratory Fitness. A modified Balke treadmill protocol was used to estimate peak oxygen uptake (VO_2_peak) [71]. Oxygen uptake was assessed using an Oxycon portable metabolic system (CareFusion, San Diego, CA, USA). ACSM guidelines were followed for test termination [72]. VO_2_peak was estimated using the Foster equation [73]. 

Aortic Pulse Wave Velocity. A certified sonographer measured aortic pulse wave velocity using the SphygmoCor XCEL system (Itasca, IL, USA). Assessment followed validated methods described in a previous publication [53]. 

Serum and Plasma Markers of Cardiometabolic Disease risk. Venous blood draws were collected after a 10 h fast. Serum and plasma were separated by centrifugation and stored at −80 °C. Plasma glucose was measured using a Cobas C-111 chemistry analyzer (Roche Diagnostics, Indianapolis, IN, USA). Triglycerides, total cholesterol, and HDL-C were assessed using a Rx Daytona chemistry analyzer (Randox Laboratories, Kearneysville, WV, USA). LDL was calculated using the Friedewald equation [74]. Pro-inflammation biomarker of tumor necrosis factor-alpha (TNF-α) was measured in serum using a commercial ELISA kit (ALPCO).

Dietary Intake. Assessed by the Arizona Food Frequency Questionnaire [75,76] and included as a covariate when examining pre-post intervention changes in BMI, blood pressure, and serum and plasma markers of cardiometabolic disease risk. Specific variables examined as potential covariates included the following: (a) total energy intake (for BMI outcomes); (b) carbohydrate intake (for triglyceride outcomes); (c) sugar intake (for insulin and fasting glucose outcomes); and (d) overall health eating index, a proxy for overall dietary quality [77], (for blood pressure and cholesterol concentration outcomes [i.e., total cholesterol, LDL, and HDL]). 

#### 2.6.5. Social Cognitive Theory Outcomes

Social Cognitive Theory constructs targeted by the intervention were assessed using previously published and validated surveys, described elsewhere [53]. These outcomes were included to provide insight on whether the intervention was successful in manipulating the PA-related psychosocial constructs it was designed to target. Constructs measured included PA-related self-efficacy (12-item Exercise Confidence Survey [78]), social support (10-item Social Support for Exercise Survey [79]), self-regulation (10-item Self-Regulation Scale from the Health Beliefs Survey [80,81]), outcome expectations (Outcome Expectation Scale for Exercise (9-items) [82]), and behavioral capability (5-item measure, adapted from previous research [83,84]). 

### 2.7. Protocol

Community-based strategies (i.e., email listservs, social media advertisements, in-person recruitment at community events) were used to recruit study participants. Interested individuals contacted the research team and were assessed for eligibility via a telephone interview or by completing an online eligibility screening survey. Eligible individuals were invited to attend an in-person orientation session to obtain more information about the study. 

At the orientation session, a trained member research team (i.e., either the study coordinator or research assistant) provided an in-depth description of all study activities, including information about the topical areas of both study arms, expectations associated with participation, study assessment procedures, and incentives for participation. At the conclusion of the orientation, written informed consent was obtained from women interested in enrolling in the study. Next, participants were given an ActiGraph GTX9 Link accelerometer to wear for 7 days. After this 7-day wear period, participants returned the accelerometer and completed an in-person study assessment where anthropometric, survey, and physiological data were collected. Participants were then randomized by the study statistician in a 1:1 allocation ratio. to receive either the *Smart Walk* or *Smart Health* intervention, as determined by an allocation list generated for a randomized permuted block design with randomly varying block sizes (of either 2 or 4 per block) using the randomizeR package [85] in R. Participants were informed of their study group assignment by either the study coordinator or research assistant. Participants were enrolled in 8 study cohorts ranging in size from 6 to 10 women over an 8 month period spanning January 2019 to August 2019. Follow-up data collection assessments were conducted at months 4 and 8 and followed similar procedures as the baseline. Technicians (i.e., phlebotomists and sonographers) conducting blood draws and aortic pulse wave velocity assessments were blinded to participants’ study group allocation at follow-up assessments. Blinding for cardiorespiratory fitness tests was not possible for all assessments, as one of the technicians conducting cardiorespiratory fitness tests also served as a research assistant for the study. However, cardiorespiratory testing procedures were standardized across participants, regardless of study arm, to limit confounding influences for this outcome. The COVID-19 pandemic resulted in 16 participants being unable to complete anthropometric and physiological study assessments (i.e., weight, blood draws, pulse wave velocity test, exercise tests) at the 8-month study timepoint because all in-person interaction with research participants was halted in March 2020. These participants were mailed a survey packet and an accelerometer to wear for 7 days with instructions to return the materials using a pre-paid self-addressed envelope. Participants received USD 50 for each study assessment; totaling USD 150 for completing all study assessments. *Smart Walk* intervention participants were allowed to keep their Fitbit after the study was over. *Smart Health* participants were provided a Fitbit device after they completed the 8-month study or at the time of intervention withdrawal (unless the participant was lost to follow-up due to non-response to research team inquiries).

### 2.8. Statistical Design 

#### 2.8.1. Sample Size Considerations

The primary aim of this study was to examine acceptability and feasibility of the intervention, rather than to formally test the intervention’s efficacy. Accordingly, the choice of sample size was selected primarily to allow for testing and refinement of protocols and procedures (Aim 1), informal examination of between-arm differences in patterns of change (Aim 2), and estimation of variability in outcomes to be used in planning a subsequent fully powered efficacy trial. We selected our sample size (N = 60 total participants; n = 30 allocated to each arm) that, based on our previous work [46,64] and statistical simulation studies regarding sample sizes for pilot trials [86], would be adequate to address these goals. As the study was not powered to test for the statistical significance of between-arm differences, our discussion of the findings (see below) focuses instead on the magnitudes of these differences (expressed as Cohen’s *d*s). In the interest of completeness, we used the FactorialPowerPlan function of the MOST package [87] in R to estimate minimum detectable effect sizes (MDESs) for a two-arm trial with (a) *n* = 30 participants per arm, (b) an ANCOVA analytic model (i.e., baseline outcome score as covariate), (c) α = 0.05, (d) power = 0.80, 0.85 or 0.90, and (e) baseline-post-test correlation of *r* = 0.30, 0.50, or 0.70. MDES estimates ranged from *d* = 0.53 (at power = 0.80 and *r* = 0.70) to *d* = 0.81 (at power = 0.90 and *r* = 0.30).

#### 2.8.2. Statistical Analyses

Univariate descriptive statistics (means, standard deviations, percentages, frequencies) were used to summarize demographic characteristics, *Smart Walk* app use, Fitbit wear, and post-intervention satisfaction survey responses. Intervention effects and effect sizes (Cohen’s *d*) were estimated using ANCOVA-type multivariable regression models predicting post-intervention (4- or 8-month follow-up) values on each outcome from study arm (intervention arm coded as 0.5, control arm coded as −0.5) and the outcome’s baseline value. Dietary intake measures were included as covariates in models for a subset of cardiometabolic outcomes. Covariate values in each model were from measurements taken at the same time as the outcome measurement (4-month or 8-month follow-up). We used Fisher’s exact tests comparing group proportions at 4- and 8-month follow-ups to examine between-group differences in meeting national PA guidelines. For self-reported PA outcomes, meeting national PA guidelines was defined by a participant reporting ≧150 min/week of MVPA according to the Exercise Vital Sign questionnaire. For accelerometer-measured PA outcomes, meeting national PA guidelines was defined, for each bout length, as having an average of ≥21.43 min/day (i.e., ≥[150 min/week]/[7 days/week]) of MVPA during the follow-up accelerometer wear period.

To mitigate bias and the loss of statistical power due to attrition, we analyzed datasets created using multiple imputation (MI). We used a fully conditional specified imputation model for each outcome to generate 150 imputed datasets in Blimp [88]. Each imputation model contained the analysis variables (study arm and baseline outcome values) and auxiliary variables that were empirically or conceptually related to the outcome scores and missingness in the outcome at follow-up. Analysis of MI data was carried out for all outcomes, except for those 8-month measures that were not collected due to COVID-19-related restrictions (e.g., anthropometrics, cardiovascular fitness, pulse wave velocity, blood biomarkers). Due to the high rate of missingness in these 8-month outcomes (ranging from 45% to 53%), analyses of MI data failed to yield sensible analytic model estimates. Accordingly, we present only the results of complete-case analyses for these measures. 

All analytic models were estimated in R [69] using the lm (“linear model”) function, and pooling of estimates from analyses of MI datasets was performed using the mitml package [89]. Effect size estimates were obtained by transforming pooled *R*^2^ values computed using the mice package [90], with 95% confidence intervals based on formulae from Morris [91].

## 3. Results

### 3.1. Participant Characteristics

Participants (n = 60) had a mean age of 38.4 (*SD* = 6.9) years, and half were either married (36.7%) or in a committed relationship (13.3%). Nearly half had obtained a bachelor’s degree (20.0%) or higher (26.7%). Income levels were diverse, with 15% reporting an annual household income of less than USD 25,000, 38.3% USD 25,000 to USD 50,000, 36.7% USD 50,001 to USD 75,000, and 10% greater than USD 75,000. Table 5 summarizes baseline characteristics for each study arm. 

### 3.2. Feasibility and Acceptability

#### 3.2.1. Recruitment and Retention

**Figure 1:**
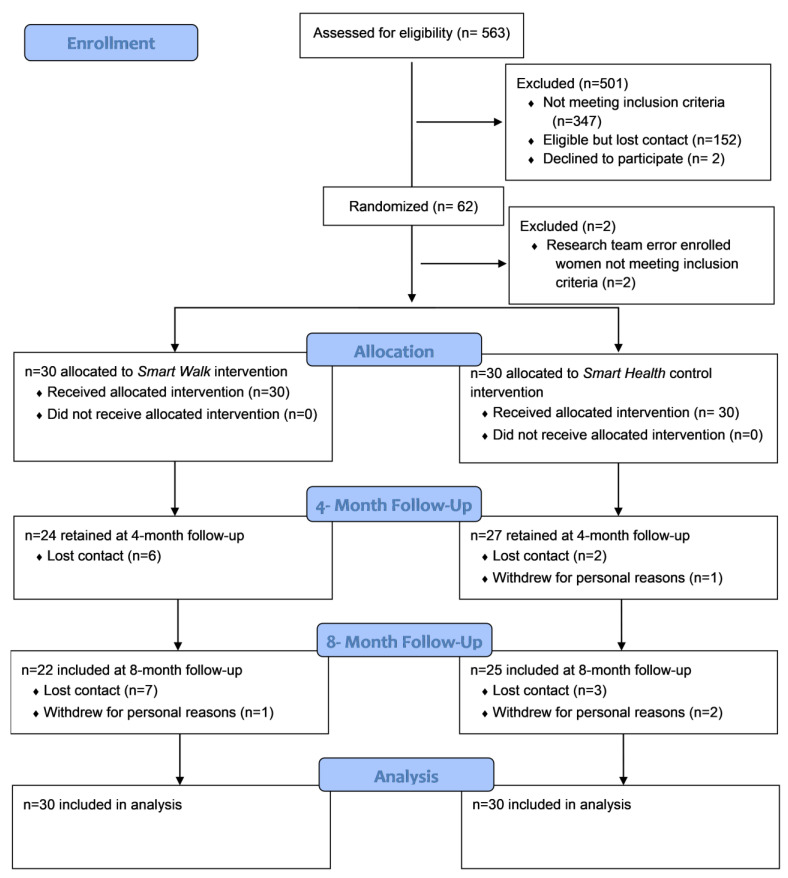
Participant flow diagram.

Figure 1 illustrates participant flow throughout the intervention. We recruited our sample (n = 60) in 8 months, equating to enrolling 7.5 women/month into the intervention. Recruitment efforts yielded in 563 women being screened for eligibility. Of these, 214 were considered eligible and 62 were allocated to receive either the *Smart Walk* PA intervention (n = 31) or the *Smart Health* comparison condition (n = 31). However, upon a routine review of study data during active recruitment, it was determined that two participants were incorrectly enrolled into the study by the research team (i.e., these participants engaged in >60 min MVPA at screening). Thus, these two participants were informed of this error and were allowed to continue to participation with acknowledgement that their data would be excluded from outcome analysis. Among the 60 eligible participants randomized, 85% (n = 51) were retained at the 4-month assessment and 78% (n = 47) at 8 months. 

#### 3.2.2. Objectively-Measured App Use, Fitbit Wear, and Participant Feedback Regarding the Intervention

**Figure 2:**
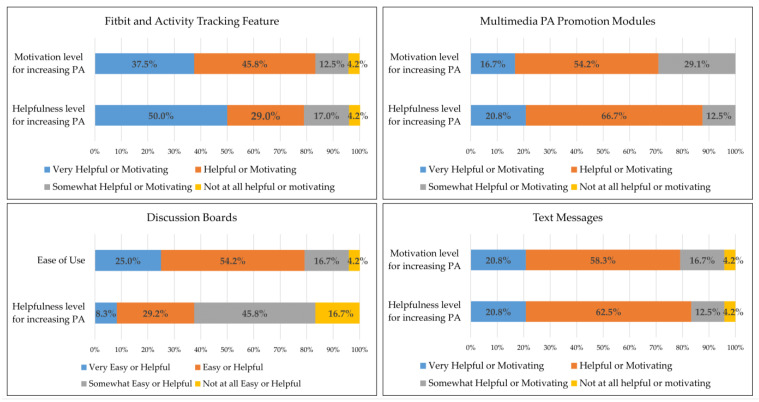
Participant-reported satisfaction with *Smart Walk* intervention components. Sums equalling greater than 100% are associated with rounding to the nearest tenth decimal place.

Fitbit Wear and Activity Tracking Feature. The 30 intervention participants, on average, wore the Fitbit on 67% of intervention days (75.4 days during the 112-day active intervention period; approximately 4.7 days/week). The 24 intervention participants retained at 4-month follow-up averaged 5.1 days/week of Fitbit wear (73% of intervention days). Fitbit wear was higher during the initial weeks of the intervention and gradually declined over time (Table 6). As illustrated in Figure 2, participant feedback regarding the Fitbit and activity tracking feature was favorable, with 96% (n = 23) of participants indicating they found these components of the study “very helpful” (n = 12), helpful” (n = 7), or somewhat helpful (n = 4) for increasing PA. Similarly, 96% of participants indicated the activity tracking components of the intervention were “very motivating” (n = 9), “motiving” (n = 11), or “somewhat motivating” (n = 3) for increasing PA. When asked about any difficulties associated with wearing the Fitbit and/or accessing the activity tracking feature, participants described forgetting to put the Fitbit back on after showering (n = 5) or washing dishes (n = 2). Some participants found the wrist placement of the device was sometimes bothersome due to a preference for not wearing things on the wrist (n = 1), the Fitbit not being visually appealing/not matching their outfit (n = 2 participants), and minor skin irritation after extended wear (n = 4).

Multimedia PA Promotion Modules. Participants, on average, viewed 7.4 of the 14 PA promotion modules at least once. Participants who completed the 4-month intervention viewed more (M = 8.2) modules non-completers (M = 4.2). Modules presented earlier in the intervention were viewed more frequently than modules that were presented later (Table 6). Post-intervention feedback from participants indicated that all participants retained at 4 months (n = 24) found the modules to be “very helpful” (n = 5), “helpful” (n = 16), or “somewhat helpful” (n = 3) for promoting PA (Figure 2). Likewise, all participants reported the modules were “very motivating” (n = 4), “motivating” (n = 13), or “somewhat motivating” (n = 7) to be physically active. When asked if they had any difficulties viewing module materials, four participants indicated intermittent issues with playing the module videos and one participant noted an issue with viewing the module text on her phone. Participants indicated these events occurred after updates to their smartphone operating system (n = 3) or when cellular phone service was poor (n = 2).

Discussion Board Feature. Engagement on the app discussion boards was modest throughout the intervention. Participants posted an average 2.1 posts per week during week 1 of the study, which declined to an average of 0.3 posts/week at week 16 (Table 6). Limited engagement on the discussion boards was corroborated by participant self-report, with 71% of participants reporting using discussion boards only once a week (n = 11) or less than once a week (n = 5). Five participants reported accessing the discussion boards 2–3 times per week, and two reported accessing them 4–6 times per week. While most participants thought the discussion boards were easy to use (Figure 2), their utility for promoting PA was limited (n = 2 “very helpful” in promoting PA, n = 7 “helpful”, n = 11 “somewhat helpful”, n = 4 “not at all helpful”). Participant narratives featured disappointment in the limited discussion board engagement among study participants (e.g., “My group didn’t participate, so within the app, I felt unsupported.”; “My group…didn’t really participate.”).

Text Messages. All PA promotion text messages (i.e., 42 messages) were delivered to 77% (n = 23) of PA intervention participants. Among the other 7 participants, text messages delivery rates ranged from 76% to 98% (32–41 messages). Patterns of non-delivery indicated temporary, participant-specific mobile phone service issues as a reason for non-delivery. Feedback indicated that nearly all (96%) participants completing the intervention reported the text messages were both helpful (n = 5 “very helpful”, n = 15 “helpful”, n = 3 “somewhat helpful”) and motivating (n = 5 “very motivating”, n = 14 “motivating”, n = 4 “somewhat motivating”; 1 participant indicated the text messages were “not at all helpful” and “not at all motivating”) for promoting PA (Figure 2).

Additional Participant Feedback Regarding Intervention Acceptability and Feasibility. Among participants completing the PA intervention (n = 24), all reported gaining knowledge about PA and exercise and that they would recommend the smartphone-delivered PA promotion program to a friend. Narratives further supported feasibility and acceptably of the intervention (e.g., “The program was great!”, “It [the study] reminded me to care for myself and that I was worth it”, “I think the program is helpful and works” and “You guys are great. Thanks for the encouragement.”) When prompted to provide feedback on how to improve the program, response focused 3 key themes emerged: (1) enhancing participant engagement on the discussion boards (e.g., “increase engagement [on the discussion boards]”, “provide facilitated [discussion board] activities”, and “encourage people to be active on the [discussion] boards.”), (2) addition of personalized feedback/counseling for PA (e.g., “one-on-coaching”, “[add] weekly or biweekly check-ins via video”), and (3) inclusion of an in-person session for participants to meet each other face-to-face (e.g., “add a face-to-face meeting” and “meet with others that are in the program as a big group”).

### 3.3. Physical Activity

The intervention group demonstrated substantially greater increases in self-reported minutes/week of MVPA compared to the comparison group at both the 4-month (model-estimated between-arm difference in change [b ± SE(b)] = 43.3 ± 17.7 min/week, *p* = 0.018; *d* = 0.69) and 8-month assessments (b = 56.6 ± 27.5 min/week, *p* = 0.046; *d* = 0.63). Group differences for change in self-reported weekly energy expenditure, regardless of intensity-level, largely mirrored self-reported MVPA outcomes (see Table 7); however, statistically significant between-group differences (at α = 0.05) were only observed for baseline to 8-month changes in light-intensity PA (b = 117.2 ± 51.9, *p* = 0.030; *d* = 0.72), moderate-intensity PA (b = 80.3 ± 35.8, *p* = 0.031; d = 0.74), and total PA (b = 309.9 ± 109.3, *p* = 0.007; *d* = 0.87). Counter to expectations, the comparison group showing increased accelerometer-measured MVPA minutes/day at 4 and 8 months, while the intervention group showed decreases a both time points; however, changes in accelerometer-measured MVPA were generally small (see Table 7).

With respect to meeting national PA guidelines, at the 4-month follow-up, 6 of 24 (25%) intervention group participants, and 3 of 27 (11%) comparison group participants self-reported engaging in ≥150 min/week of MVPA (odds ratio [*OR*] = 2.62, 95% CI = [0.48, 18.36], *p* = 0.276). At 8 months, 6 of 22 (27%) intervention group participants and 2 of 25 (8%) of comparison group participants met the 150 min/week recommendation (*OR* = 4.18, 95% CI = [0.64, 47.51], *p* = 0.123). Among participants providing accelerometer data at 4 months, 5 of 23 (22%) in the intervention group and 8 of 22 (36%) in the comparison group averaged > 21.4 min/day of MVPA according to 1 min bouts (*OR* = 0.49, 95% CI = [0.10, 2.17], *p* = 0.337). Based on a 10 min bout length, no intervention participants and only 1 control group participant met threshold (*OR* = 0.00, 95% CI = [0.00, 37.30], *p* = 0.489). At 8 months, 3 of 12 intervention participants (25%) and 3 of 15 comparison participants (20%) met this threshold according to 1 min bouts (*OR* = 1.24, 95% CI = [0.14, 11.05], *p* = 1.000). No participants met this threshold at 8 months based on a 10 min bout length.

### 3.4. Cardiometabolic Risk Markers

Cardiometabolic risk outcomes are presented in Table 8. Between-arm differences in change from baseline to 4 months were clinically relevant, but not statistically significant, with intervention participants showing greater improvements in cardiorespiratory fitness (b = 1.67 ± 0.96 mL/kg/min, *p* = 0.090; *d* = 0.40), systolic blood pressure (b = −3.33 ± 3.84 mmHg, *p* = 0.391; *d* = 0.22), diastolic blood pressure (b = −4.28 ± 3.14 mmHg, *p* = 0.179; *d* = 0.37), and pulse wave velocity (b = −0.46 ± 0.29 m/s, *p* = 0.131; *d* = 0.33; see Table 8). Differences in baseline to 4-month change for BMI and lipid, serum, and inflammatory markers of cardiometabolic disease risk (total cholesterol, HDL, LDL, triglycerides, glucose, insulin, TNF-α) were negligible, regardless of study arm, with no significant between-group differences in change. Analyses of multiply imputed datasets for 8-month cardiometabolic disease risk outcomes yielded implausible estimates; accordingly, we present results from complete-case analyses for those change in those outcomes (Table 8).

### 3.5. Social Cognitive Theory Outcomes

As illustrated in Table 9, between-group differences in change in behavioral capability scores were large and statistically significant at 4 months (b = 1.44 ± 0.35, *p* < 0.001: *d* = 1.22) and 8 months (b = 1.33 ± 0.48, *p* = 0.009; *d* = 0.92). Differences in change from baseline to 4 months in PA self-efficacy (b = 0.28 ± 0.18, *p* = 0.130; *d* = 0.39,), social support from family (b = 4.07 ± 3.57, *p* = 0.260; *d* = 0.31) and PA self-regulation (b = 0.22 ± 0.22, *p* = 0.319; *d* = 0.29), were small to moderate in magnitude, with the intervention group demonstrating greater (but not statistically significant) improvements on these constructs relative to controls. Baseline to 8-month changes in social support from family (b = 4.00 ± 4.03, *p* = 0.326; *d* = 0.27) were similar to those observed at 4 months. Self-efficacy declined in both groups from baseline to 8 months, with only a negligible between-group difference in change (b = −0.09 ± 0.26, *p* = 0.723; *d* = −0.17). From baseline to 8 months, both groups showed increased self-regulation scores, with only small between-group difference in change (b = 0.23 ± 0.30, *p* = 0.438; *d* = 0.28). Outcome expectations and social support from friends showed negligible change in both groups at 4 and 8 months.

## 4. Discussion

The current study examined the acceptability and feasibility of a smartphone-delivered PA intervention for AA women and explored between-group differences for patterns of change in cardiometabolic disease risk outcomes and hypothesized theoretical mediators. Overall, results for the feasibility and acceptability of the intervention were favorable, while also affording insight into how the intervention could be refined prior to implementation of a fully powered RCT. Observed patterns of change in self-reported MVPA and several cardiometabolic disease outcomes, including cardiorespiratory fitness and blood pressure, further demonstrate the promise of our smartphone-delivered PA intervention. These encouraging findings make an important contribution to the limited body of research examining the utility of mHealth approaches in increasing PA and improving cardiometabolic health among AA women.

### 4.1. Feasibility and Acceptability

Feasibility and acceptability of the intervention were supported by numerous indices, including participant recruitment and retention, results of the post-intervention satisfaction survey, and app use. Recruitment efforts yielded an enrollment rate of 7.5 women/month. Although comparison of this enrollment rate to other studies has limited utility due to the heterogeneity that exists across studies in regards to study populations, implementation settings, recruitment methods, and inclusion/exclusion criteria, this rate will be used to develop recruitment timelines for future intervention trials of the *Smart Walk* intervention. The high retention rates at the end of the active 4-month intervention (85%) and at the 8-month follow-up (78%) are comparable to median rates of studies included in previous reviews of PA interventions for AA women (i.e., 83%) [35], including mHealth interventions (i.e., 80%) [43]. To our knowledge, this was the first mHealth PA intervention among AA women to include a delayed post-intervention follow-up to examine longer-term maintenance of PA, further highlighting the effectiveness of our retention strategies.

Participants expressed a high level of satisfaction for the multi-media modules, text message, and Fitbit components of the mHealth intervention, as well as for the intervention as a whole, with all participants who completed the intervention indicating they would recommend the smartphone-delivered PA intervention to a friend. These satisfaction results mirror those of an earlier iteration of the structured PA intervention (i.e., delivered through Facebook and text messages) [46] and of a pilot test of the *Smart Walk* intervention among AA women aged 50–65 years [48], suggesting our mHealth PA intervention efforts continue to be well-received. Though largely satisfied with the intervention, lack of pre- to post-intervention change in reports of social support, limited discussion forum participation, and participant feedback on the satisfaction survey strongly indicated a need to enhance social support provided within the intervention. Among our unique sample of AA women with obesity and limited history or PA engagement, a more facilitated and individualized approach may be needed to enhance social support for PA. Our findings also support extant literature on the importance of social support for PA promotion among AA women [37,58,92]. For future interventions, we plan to incorporate lay community health workers to serve as virtual PA coaches to actively engage with participants on the discussion forums and provide weekly, individualized PA coaching/counselling sessions via commercially available videoconferencing platforms (i.e., FaceTime, Google Hangouts).

App use and Fitbit wear data revealed that engagement was high during the initial weeks of the intervention, but declined gradually over the course of the intervention. Although decreased engagement over time is commonly reported in e- and mHealth interventions [93], it represents another important aspect to address in future interventions. Building upon participant feedback and recent publications describing best practices to promote mHealth user engagement [94,95,96], strategies we intend to explore in future research include: (a) refining the smartphone app and study protocols to include additional push notifications encouraging app engagement and Fitbit wear (e.g., encouraging participants to access unviewed PA promotion modules and to wear/sync the Fitbit if no data have been received by the research team in a 24 h period), (b) incorporating PA coaches to actively engage and foster dialogue on the app discussion boards and reinforce weekly module content through individualized PA coaching, and (c) having an in-person “kick-off” session among participants prior to engaging virtually on the *Smart Walk* app. We anticipate these strategies collectively will promote app engagement and foster a greater sense of social support for PA among study participants.

### 4.2. Physical Activity and Cardiometabolic Disease Risk Outcomes

Intervention participants self-reported greater MVPA increases than comparison group participants at both post-intervention assessments. Changes in self-reported energy expenditure followed a similar pattern. Self-reported MVPA changes from baseline to 4 months are comparable to improvements observed in a previous e- and mHealth PA intervention with AA women [43] and those observed across previous mHealth PA interventions among predominately White populations (36–73 min/week) [40,41,42]. The finding that participants continued to increase self-reported PA from 4 to 8 months (i.e., after removal of the active intervention) is promising, as self-reported improvements in PA usually attenuate or decline at distal post-intervention follow-up assessments [41]. Despite improvements in self-reported activity, we failed to observe increases in accelerometer-measured MVPA. Measurement error may have contributed to these counterintuitive results. Activity levels were classified using the only publicly available MVPA cut points for non-dominant hand wrist-worn accelerometers [68,70], which were developed using a convenience sample of healthy and normal weight Norwegian adults. These cut points are likely too high to accurately assess MVPA among our study population of AA women with obesity and limited history of PA engagement. Future research is needed to derive appropriate wrist-worn accelerometer cut points for women who are both insufficiently active and with obesity.

Meaningful, though not statistically significant, improvements in cardiorespiratory fitness and blood pressure were observed among intervention participants. The descriptive increase in fitness among PA intervention participants (increase of 1.1 mL/kg/min vs. a decrease of 0.8 mL/kg/min in comparison group) corresponds to a 7–13% decrease in risk for premature mortality [97]. Likewise, intervention participants showed descriptively greater (though not statistically different) improvements in blood pressure as compared to the control group. Mean improvements in blood pressure among *Smart Walk* participants (i.e., −3.8 mm Hg for systolic blood pressure and −4.3 mm Hg for diastolic blood pressure) are comparable to those reported by meta-analyses examining the impact of aerobic exercise training on blood pressure outcomes [98,99]. Such improvements are considered clinically relevant, as 3–5 mm Hg improvements in systolic and diastolic blood pressure are associated with decreased risk of cardiovascular disease, stroke, and premature death [100,101].

Observed patterns of change for aortic pulse wave velocity, a measure of arterial stiffness and independent predictor of cardiovascular events and mortality [102,103], were also greater for intervention participants than for comparison group participants. Improvement among intervention participants (decrease of 0.3 m/s) were slightly smaller than that reported in a meta-analysis examining the role aerobic exercise for improving pulse wave velocity among adults with baseline values comparable to those of our sample [104,105]. Previous studies examining the impact of aerobic PA on pulse wave velocity have predominately involved supervised, structured exercise sessions at a closely monitored moderate-to-vigorous intensity levels [104,105]. Few community-based studies have included this measure, where participants likely, on average, engage in PA for shorter bouts and at lower intensity levels. We encourage wider use of this measure in future studies to help build a clearer understanding of how mHealth PA interventions may improve arterial stiffness.

BMI was unchanged throughout the intervention. This outcome was not surprising, as weight loss from PA alone requires participants to engage in substantially more MVPA than levels promoted by the study (i.e., >225 min/week) [106,107]. Glucose and lipid outcomes also showed minimal between group changes, which were largely within the margin of measurement error and within ranges expected for day-to-day variability. This finding was somewhat surprising given the positive trends observed for cardiorespiratory fitness, blood pressure, and pulse wave velocity. Several factors may have contributed to this outcome, including participants having relatively normal levels for these outcomes at baseline, which offered limited room for improvement, inadequate fasting prior to the blood draw assessment (i.e., less than 10 h), and/or participants failing to achieve PA levels necessary to improve these outcomes. The mixed results for these blood-derived markers of cardiometabolic disease risk are hypothesis generating and should be explored in future research.

### 4.3. Psychosocial Outcomes

Substantially greater improvement in behavioral capability for PA for intervention participants suggests that the intervention curriculum was successful in enhancing knowledge of national PA guidelines, the types of activities that constitute aerobic PA, and the health benefits of aerobic PA. We also observed small-to-moderate, albeit not statistically significant, baseline to 4-month increases in self-efficacy and self-regulation among intervention participants relative to comparison group participants. While promising, these finding highlight the need to further enhance the theoretical fidelity [108] of intervention components targeting these constructs, which are important to both adoption and maintenance of PA [109,110]. Small, but non-significant, changes in social support from family were reported, while social support from friends was relatively unchanged. Given that social support is a key psychosocial process associated with PA engagement among AA women, identifying effective mHealth intervention strategies to enhance social support may be key to producing even greater improvements in PA. Lastly, outcome expectations for PA remained unchanged throughout the study, which may reflect a ceiling effect, as participants reported strongly positive expectations at baseline, affording only limited room for improvement.

### 4.4. Limitations, Strengths, and Future Directions

The study’s findings are encouraging, but it is important to recognize potential limitations. The primary focus for this stage of intervention development was on feasibility and acceptability of the smartphone-delivered intervention, rather than efficacy testing. Accordingly, when interpreting PA, cardiometabolic, and psychosocial outcomes, we focused on examining magnitudes of effect, rather than statistical significance. Results for these outcomes (and our subsequent discussion of them) should be interpreted as suggestive, rather than definitive. Additionally, the multi-component nature of the intervention limits our ability to determine if one component of the intervention may have been more strongly related to changes in PA behavior than another. To properly address this type of question, a design that can examine component-specific effects is needed (e.g., factorial or multiphase optimization strategy [MOST] designs). A third limitation was that although efforts were made to blind research technicians conducting cardiorespiratory fitness tests to participants’ study group allocation at 4- and 8-month follow-up assessments, this was not always possible. Standardized testing procedures were used to limit this potential bias. Other limitations include recruiting participants from single geographical area (i.e., Phoenix, Arizona), using community-based recruitment methods, and providing compensation for participation (although this compensation was nominal compared to study time commitments). These considerations may limit generalizing results to AA women outside of the Phoenix metropolitan area and likely reflect a sample with increased motivation to adopt healthy lifestyle behaviors. Finally, restrictions on data collection related to the COVID-19 pandemic had serious impacts on conclusions that could be drawn from analyses of several 8-month outcomes. The lack of 8-month physiological study assessments for approximately 1/3 of our sample resulted in untrustworthy estimates from analyses of multiply imputed datasets, limiting us to complete-case analyses of cardiometabolic outcomes at 8 months. These restrictions also likely influenced PA behaviors and psychosocial outcomes among participants who were completing the study during the early stages of the pandemic.

Despite limitations, numerous strengths of the study should also be noted. This is one of the first studies to examine the effects of a culturally tailored smartphone-delivered PA intervention among AA women, research necessary to advance e- and mHealth research on PA promotion. Second, we addressed several design limitations of previous studies [43], by including a comparison group, deep structure culturally tailored intervention activities, objective measurement of PA, and a delayed post-intervention follow-up assessment to assess longer-term effects of the intervention. We also included both traditional and novel markers of cardiometabolic disease risk, which few e- or mHealth studies of PA interventions have examined. These design characteristics enhance the scientific rigor of the e- and mHealth PA intervention research for AA women.

Future directions for this program of research include refining the *Smart Walk* intervention to enhance usability, engagement, and theoretical fidelity to Social Cognitive Theory constructs hypothesized to mediate intervention effects on PA outcomes prior to larger-scale efficacy testing. Specific refinements will include: (i) holding an in-person “kick-off” sessions at the beginning of the study for each cohort to further create a sense of community among participants prior to engaging virtually on the app; (ii) including additional app push notifications to prompt Fitbit wear (if needed) and for participants to view unviewed multi-media modules and discussion board posts, and (iii) incorporating lay community health coaches to actively facilitate participant engagement on the discussion boards and to provide virtual PA coaching sessions (i.e., telephone or videoconference) to study participants.

## 5. Conclusions

This study demonstrated high acceptability and feasibility, and yielded promising findings regarding the intervention’s associations with increased PA and reduced cardiometabolic disease risk among insufficiently active AA women with obesity.

## Figures and Tables

**Table 1:** Module topics for the *Smart Walk* and *Smart Health* study groups.

		Module Topic	
Week Number	Module Number	*Smart Walk* Physical Activity Intervention	*Smart Health* Comparison Intervention
1	1	Overview of the health benefits of PA and the national PA guidelines	Healthy Skin Care Practices
2	2	PA-related health disparities among AA women; Importance being a PA role model in the AA community	Hydration and Water Consumption
3	3	PA time management	Heat Illness: Symptoms and Prevention
4	4	PA goal setting	Communicating with Doctor
5	5	Overcoming general barriers to PA	Breast Cancer: Prevention and Screening
6	6	Tips for increasing daily PA	Cervical Cancer: Prevention and Screening
7	7	Overcoming hair-related barriers to PA	Hair Care
8	8	Social support for PA	Oral Health
9	9	Trying new types of activities	Vision and Eye Health
10	10	Reducing sedentary time	Sickle Cell Disease
11	11	Healthy eating complement PA	Stress Management
12	12	Muscle-strengthening and stretching activities	Cold and Flu Prevention
13	N/A		
14	13	Dealing with PA setbacks.	Tips to Stay Healthy when Travelling
15	N/A		
16	14	Review of previous modules and PA maintenance	Review of Previous Modules

Abbreviation: PA = physical activity.

**Table 3:** Deep structure cultural considerations.

Cultural Consideration	Description	How the Cultural Consideration Was Incorporated into the Intervention
Collectivism	Attending to the needs and well-being of others over the needs and well-being of one’s self. This can contribute to AA women reporting lack of time, energy, or resources for PA. Notably, this consideration is reported among women regardless of races/ethnicities, but previous research suggests this concept may be more accentuated in the AA community.	PA promotion materials: Acknowledged caretaking as integral to the value system of many AA women. Promoted PA as an investment in one’s health and well-being of Emphasized PA should not be viewed as a competing interest for one’s caretaking responsibilities. Encouraged PAto be viewed as a behavior to help participants achieve their caretaking, familial, and community responsibilities with more energy and for a longer duration throughout the lifespan.
Racial pride/Role modelling	Awareness and interest in how one’s behavior can contribute the collective health and well-being of the AA community.	Intervention materials underscored the notion that physically active AA women are positive role models, which can encourage other members of the AA community to be physically active
Physical appearance preferences	Some AA women are hesitant to engage in PA because: Sweat negatively impacts their hairstyle. They perceive PA will alter their desired body shape.	Intervention materials: Described strategies to help reduce the negative effects of sweat on hairstyles Encouraged adoption of hair styles less effected by sweat (i.e., braids, natural hairstyles). Informed participants that meeting national aerobic PA guidelines (i.e., 150 min/week) does not substantially change the body shape of most women unless dietary changes are also made. Underscored the health benefits of PA independent of weight loss, reduced risk for heart disease, type 2 diabetes, increased energy).

**Table 4:** Comparison of *Smart Walk* and *Smart Health* intervention components.

Intervention Component	*Smart Walk* Intervention	*Smart Health* Intervention
**Features available on the smartphone application**		
Personal profiles	X	X
Weekly multi-media modules	X	X
Discussion boards	X	X
Activity tracker that integrates with the Fibit device	X	
**Text messages delivered 3 times per week**	X	X

**Table 5:** Summary and comparisons of participant demographic characteristics by study arm.

Characteristic	*Smart Walk*	Comparison
Age (years; *M* and *SD*)	37.2 (7.0)	39.5 (6.8)
Relationship status (*n* and %)		
Married	10 (33%)	12 (40%)
Committed partnership	6 (20%)	2 (7%)
Divorced	6 (20%)	3 (10%)
Separated	0 (0%)	1 (3%)
Never married	8 (27%)	12 (40%)
Number of children < 18 years (Median and range)	1 (0–5)	1 (0–4)
Education (*n* and %)		
Some high school	1 (3%)	0 (0%)
High school diploma or GED	5 (17%)	2 (7%)
Some college or technical school	13 (43%)	11 (37%)
Bachelor’s degree	5 (17%)	7 (23%)
Master’s degree	5 (17%)	10 (33%)
Doctoral degree	1 (3%)	0 (0%)
Annual income (*n* and %)		
<USD 25 K	6 (20%)	3 (10%)
USD 25,001–USD 50 K	12 (40%)	11 (37%)
USD 50,001–USD 75 K	10 (33%)	12 (40%)
USD 75,001–USD 100 K	2 (7%)	3 (10%)
>USD 100 K	0 (0%)	1 (3%)
Employment status (*n* and %)		
Employed, Part-time	6 (20%)	4 (14%)
Employed, Full-time	19 (63%)	24 (83%)
Currently unemployed	4 (13%)	2 (7%)

**Table 6:** *Smart Walk* App engagement and Fitbit wear among participants allocated to the PA intervention (n = 30).

	Module/Intervention Week ^a^															
Outcome	1	2	3	4	5	6	7	8	9	10	11	12	13	14	15	16
Mean # of module views/participant	2.5	2.3	1.8	1.2	1.2	1.0	1.1	0.9	0.8	1.0	0.6	0.7	n/a	0.9	n/a	0.3
Mean # of discussion posts/participant	2.1	1.1	1.1	0.8	0.3	0.6	0.4	0.4	0.9	0.4	0.4	0.4	n/a	0.4	n/a	0.3
Mean # Fitbit wear days/week/participant ^b^	5.7	5.7	5.9	5.5	5.3	4.7	4.9	4.7	4.7	4.4	4.1	3.7	3.5	3.7	4.0	3.9

^a^ Module views/discussion board post data are not presented for weeks 13 and 15, as no new modules/discussion boards are posted these weeks. ^b^ Fitbit wear criterion, based on work by Hartmann et al. [63], required at least 1 min of light- or moderate PA/day.

**Table 7:** Physical Activity Outcomes.

		Baseline	4-Month	8-Month	Baseline to 4-Month Change		Baseline to 8-Month Change	
PA Outcome	Study Arm	M (SD)	M (SD)	M (SD)	*d*^c^ (95% CI)	*p* ^e^	*d*^c^ (95% CI)	*p* ^e^
Self-reported MVPA (min/week) ^a^	*Smart Walk*	30.7 (37.3)	102.2 (73.5)	114.1 (116.1)	0.69	0.018	0.63	0.046
	Comparison	30.0 (43.8)	58.5 (56.6)	56.9 (74.8)	(0.16, 1.22)		(0.10, 1.16)	
Self-reported Estimated Energy Expenditure (MET-min/week) ^b^								
Light PA	*Smart Walk*	79.1 (187.6)	266.2 (436.2)	171.1 (218.1)	0.41	0.159	0.72	0.030
	Comparison	77.0 (109.6)	131.4 (199.5)	53.6 (89.1)	(−0.11, 0.93)		(0.19, 1.25)	
Moderate PA	*Smart Walk*	81.7 (234.9)	222.5 (363.6)	95.2 (131.0)	0.50	0.090	0.74	0.031
	Comparison	17.7 (35.0)	80.0 (168.2)	17.6 (88.6)	(−0.02, 1.02)		(0.21, 1.27)	
Vigorous PA	*Smart Walk*	80.5 (128.2)	324.2 (514.1)	247.8 (228.5)	0.38	0.196	0.60	0.052
	Comparison	100.8 (139.8)	183.2 (309.6)	128.4 (181.3)	(−0.14, 0.90)		(0.07, 1.13)	
Total PA	*Smart Walk*	241.3 (500.4)	871.9 (1065.9)	524.9 (437.5)	0.53	0.074	0.87	0.007
	Comparison	410.2 (1169.6)	396.1 (554.9)	212.2 (275.9)	(0.01, 1.05)		(0.33, 1.41)	
Accelerometer-measured MVPA (min/day)								
1 min bouts	*Smart Walk*	16.9 (13.1)	13.8 (21.4)	15.5 (16.0)	−0.39	0.202	−0.41	0.078
	Comparison	13.4 (8.1)	20.0 (15.9)	14.6 (8.1)	(−0.91, 0.13)		(−0.93, 0.11)	
10 min bouts	*Smart Walk*	3.4 (7.1)	1.3 (4.9)	1.4 (4.3)	−0.25	0.536	−0.25	0.430
	Comparison	1.0 (2.9)	1.4 (5.0)	1.3 (2.2)	(−0.77, 0.27)		(−0.77, 0.27)	

^a^ Assessed by the Exercise Vital Sign. ^b^ Assessed by the RECIGOR Short Physical Activity Questionnaire. ^c^ Cohen’s *d* effect size estimate. ^e^ Probability value (i.e., *p*-value).

**Table 8:** Cardiometabolic Disease Risk Outcomes.

		Baseline	4-Month	8-Month	Baseline to 4-Month Change		Baseline to 8-Month Change	
PA Outcome	Study Arm	M (SD)	M (SD)	M (SD)	*d*^e^ (95% CI)	*p* ^f^	*d*^e^ (95% CI)	*p* ^f^
BMI (kg/m^2^) ^a^	*Smart Walk*	40.0 (6.7)	39.2 (6.6)	36.3 (9.9)	−0.14	0.147	0.27	0.317
	Comparison	41.2 (7.3)	41.3 (7.5)	41.2 (7.0)	(−0.66, 0.38)		(−0.49, 1.03)	
Systolic Blood Pressure (mmHG)	*Smart Walk*	134.6 (14.6)	130.8 (17.2)	130.2 (20.3)	−0.22	0.391	−0.01	0.977
	Comparison	136.2 (15.8)	135.2 (16.1)	134.1 (15.6)	(−0.74, 0.30)		(−0.78, 0.76)	
Diastolic Blood Pressure (mmHG)	*Smart Walk*	83.4 (11.8)	79.1 (13.5)	81.8 (9.6)	−0.37	0.179	−0.16	0.602
	Comparison	84.6 (10.9)	84.0 (11.3)	85.2 (9.0)	(−0.89, 0.15)		(−0.93, 0.61)	
Cardiorespiratory Fitness (mL/kg/min)	*Smart Walk*	18.8 (2.9)	19.8 (4.7)	18.3 (2.9)	0.40	0.090	−0.17	0.518
	Comparison	18.5 (3.7)	17.8 (4.2)	17.4 (2.9)	(−0.12, 0.92)		(−0.98, 0.64)	
Pulse wave velocity (m/s)	*Smart Walk*	7.7 (1.4)	7.4 (1.6)	7.3 (1.1)	−0.33	0.131	−0.01	0.976
	Comparison	7.2 (1.0)	7.3 (1.3)	7.1 (1.0)	(−0.85, 0.19)		(−0.78, 0.76)	
Total cholesterol (mg/dL) ^b^	*Smart Walk*	184.4 (39.2)	193.2 (44.9)	186.8 (50.4)	0.13	0.986	0.18	0.364
	Comparison	188.6 (29.4)	197.8 (40.4)	192.2 (35.2)	(−0.39, 0.65)		(−0.63, 0.99)	
LDL cholesterol (mg/dL) ^c^	*Smart Walk*	119.9 (33.7)	124.5 (36.9)	117.2 (38.6)	−0.11	0.878	0.27	0.253
	Comparison	122.2 (27.0)	128.6 (31.5)	127.7 (26.8)	(−0.63, 0.41)		(−0.54, 1.08)	
HDL cholesterol (mg/dL) ^c^	*Smart Walk*	46.6 (8.4)	50.2 (12.6)	50.3 (12.1)	0.25	0.336	0.00	0.982
	Comparison	48.5 (11.3)	48.9 (14.0)	48.7 (11.1)	(−0.27, 0.77)		(−0.80, 0.80)	
Triglycerides (mg/dL) ^c^	*Smart Walk*	88.6 (37.5)	91.9 (35.4)	96.3 (40.2)	−0.12	0.873	0.06	0.213
	Comparison	89.2 (51.4)	90.6 (42.7)	78.9 (39.4)	(−0.64, 0.40)		(−0.74, 0.86)	
Fasting glucose (mg/dl) ^d^	*Smart Walk*	107.5 (54.6)	112.0 (46.4)	100.0 (8.9)	0.14	0.402	0.06	0.858
	Comparison	107.4 (29.4)	106.9 (29.2)	108.2 (51.0)	(−0.38, 0.66)		(−0.74, 0.86)	
Insulin (µIU/mL) ^d^	*Smart Walk*	20.9 (18.8)	21.3 (28.0)	27.4 (18.7)	0.20	0.916	0.40	0.324
	Comparison	22.8 (17.2)	20.9 (15.6)	23.8 (16.4)	(−0.32, 0.72)		(−0.41, 1.21)	
TNF-α (pg/mL)	*Smart Walk*	1.5 (1.5)	1.6 (1.8)	1.1 (0.5)	0.08	0.361	−0.16	0.439
	Comparison	1.3 (0.5)	1.3 (0.5)	1.4 (0.6)	(−0.44, 0.60)		(−0.97, 0.65)	

Notes: ^a^ adjusted for total energy intake. ^b^ adjusted for carbohydrate intake. ^c^ adjusted for Healthy Eating Index score. ^d^ adjusted for sugar intake. ^e^ Cohen’s *d* effect size estimate. ^f^ Probability value (i.e., *p*-value).

**Table 9:** Social Cognitive Theory Outcomes.

			Baseline	4-Month	8-Month	Baseline to 4-Month Change		Baseline to 8-Month Change	
SCT Construct	Range ^a^	Study Arm	M (SD)	M (SD)	M (SD)	*d*^b^ (95% CI)	*p* ^c^	*d*^b^ (95% CI)	*p* ^c^
PA Behavioral Capability	1–5	*Smart Walk*	1.5 (1.0)	3.1 (1.5)	2.9 (1.7)	1.25	<0.001	0.92	0.009
		Comparison	1.7 (0.8)	1.8 (0.9)	1.6 (1.4)	(0.68, 1.82)		(0.38, 1.46)	
Exercise Self-Efficacy	1–5	*Smart Walk*	4.0 (0.7)	4.1 (0.8)	3.6 (1.0)	0.39	0.130	−0.17	0.723
		Comparison	3.9 (0.6)	3.8 (0.7)	3.6 (0.8)	(−0.13, 0.91)		(−0.69, 0.35)	
PA Self-regulation	1–5	*Smart Walk*	2.2 (0.9)	2.7 (0.8)	2.5 (1.1)	0.29	0.319	0.29	0.438
		Comparison	2.1 (0.5)	2.5 (0.9)	2.3 (1.0)	(−0.23, 0.81)		(−0.23, 0.81)	
Social Support for PA from Family	10–50	*Smart Walk*	24.9 (9.9)	30.7 (15.9)	31.4 (19.7)	0.31	0.260	0.27	0.326
		Comparison	22.3 (9.6)	25.1 (11.3)	24.8 (12.3)	(−0.21, 0.83)		(−0.25, 0.79)	
Social Support for PA from Friends	8–40	*Smart Walk*	20.3 (9.8)	21.3 (12.4)	21.2 (13.4)	0.12	0.879	−0.17	0.750
		Comparison	19.3 (8.6)	20.0 (10.3)	21.5 (10.7)	(−0.31, 0.73)		(−0.69, 0.35)	
Outcome Expectations	1–5	*Smart Walk*	4.2 (0.5)	4.3 (0.6)	4.0 (1.0)	0.34	0.260	−0.17	0.868
		Comparison	4.2 (0.4)	4.1 (0.5)	4.0 (0.7)	(−0.18, 0.86)		(−0.69, 0.35)	

Notes: ^a^ Potential range of scores for each survey measures. ^b^ Cohen’s *d* effect size estimate. ^c^ Probability value (i.e., *p*-value).

## Data Availability

The data presented in this study are available on request from the corresponding author.
